# Mobile cognitive testing captures divergent longitudinal trajectories of verbal learning in adults with and without HIV

**DOI:** 10.3389/fdgth.2026.1792496

**Published:** 2026-06-16

**Authors:** Alena Stasenko, Laura M. Campbell, Anne Heaton, Emily W. Paolillo, David J. Moore, Robert K. Heaton, Colin A. Depp, Amy Pinkham, Robert A. Ackerman, Philip D. Harvey, Raeanne C. Moore

**Affiliations:** 1 Department of Radiation Medicine & Applied Sciences, UC San Diego, United States; 2 Department of Psychiatry, UC San Diego, United States; 3 Edward and Pearl Fein Memory and Aging Center, University of California, San Francisco, United States; 4 Department of Psychology, School of Behavioral and Brain Sciences, The University of Texas at Dallas, Dallas, United States; 5 Psychiatry and Behavioral Sciences, University of Miami, Miami, United States

**Keywords:** aging, HIV—human immunodeficiency virus, mobile cognitive assessment, remote cognitive assessment, verbal learning and memory

## Abstract

**Introduction:**

Advanced cognitive aging remains a major concern for people living with HIV (PWH), even in the context of viral suppression. This underscores the need for sensitive tools that can detect subtle cognitive change. Mobile cognitive assessments offer a scalable and ecologically valid approach, yet their sensitivity to longitudinal change in clinical populations is not well established.

**Methods:**

We examined longitudinal performance and predictors of change on a 14-day mobile Verbal Learning Test (mVLT) administered remotely at baseline and again 12–46 months (*M* = 26.7) later in 24 PWH and 13 HIV-negative controls aged 51–74, and compared these trajectories with change on standard in-person neuropsychological testing.

**Results:**

Aggregate mean mVLT performance improved over time among controls, but this was not evident among PWH (i.e., a significant group X time interaction). In contrast, longitudinal trajectories did not differ by group on the standard in-person Hopkins Verbal Learning Test-Revised, suggesting greater sensitivity of the mobile measure in this sample. Age moderated mVLT trajectories, such that increasing age was associated with worse longitudinal trajectories in PWH, whereas age was unrelated to change in controls. Among PWH, worse mVLT trajectories were associated with higher cerebrovascular risk, lower social functioning, and poorer baseline global learning performance, but not depressive symptoms, HIV disease markers, or other medical comorbidities.

**Discussion:**

These preliminary findings suggest that the mVLT captures group-level differences in longitudinal learning trajectories and heterogeneity in performance over time among PWH, in line with contemporary models of cognitive aging in HIV. With replication in larger samples, mobile assessments could support scalable monitoring of cognitive function in PWH.

## Introduction

The population of adults with human immunodeficiency virus (HIV) who are reaching advanced ages is rapidly expanding (1), with more than half of people living with HIV (PWH) now aged 50 or older (2). Despite effective viral suppression with antiretroviral therapy (ART), older PWH remain vulnerable to neurocognitive risks due to the combined effects of aging, chronic inflammation, potential toxicity from long-term ART exposure, and a greater burden of medical comorbidities. These factors have led to proposals of *accelerated* or *accentuated* aging (3–5) and concerns about increased risk for dementia in PWH (6).

Although HIV-Associated Neurocognitive Disorders (i.e., HAND) (7, 8) are often characterized as non-progressive, several longitudinal studies demonstrated cognitive and brain health decline in both younger and older PWH (9–11). For example, episodic learning and memory—one of the most commonly impaired domains in HAND—showed age-accelerated decline in older PWH (10). Decline in global cognition over more than a decade was also reported even in younger age groups (9), which was associated with greater medical, psychiatric, and cerebrovascular comorbidity burden, and not with HIV disease or treatment-related variables (9). In addition, progressive brain atrophy has been observed even in well-treated older PWH with adequate viral suppression (12), pointing to a general CNS vulnerability.

Given this evidence, sensitive tools capable of detecting subtle within-person cognitive change are needed to monitor cognitive health and identify individuals at heightened risk for decline. Mobile, remote cognitive assessments offer a scalable, valid, low-burden approach for repeated measurement in naturalistic environments (13–15). Compared to single session in-person assessment, repeated mobile testing may offer greater sensitivity to longitudinal change by aggregating performance across multiple daily administrations, reducing measurement noise due to day-to-day variability and transient factors, while capturing learning efficiency across naturalistic daily settings rather than a single structured laboratory visit (13, 16). However, data supporting their sensitivity to longitudinal cognitive change in various clinical populations remain limited. Our group recently developed and validated a mobile Verbal Learning Test (mVLT), showing that it captures HIV-related differences in verbal learning and corresponds closely with gold-standard neuropsychological tests (17). Because the word lists lack semantic structure, the mVLT minimizes the benefit of organizational encoding strategies, placing greater demands on episodic learning relative to semantically structured list-learning tasks. In addition, a version of this test implemented within the NeuroUX platform has been shown to differentiate normal aging from those with mild cognitive impairment in older adults (18) and varying degrees of cognitive impairment between bipolar disorder (19) and schizophrenia (20). However, whether the mVLT can detect meaningful longitudinal cognitive change and differentiate trajectories in PWH from those of age-matched peers remains unknown.

This study had two primary aims. First, we evaluated changes in mVLT performance over approximately two years in PWH vs. HIV-negative controls and compared this to change on a gold-standard lab-based verbal learning measure. Second, to test whether older age makes PWH more vulnerable to accelerated cognitive aging-related change, we examined interactions between age and HIV status on mVLT trajectories. Within the PWH group, we also examined whether baseline cerebrovascular risk, overall medical comorbidity burden, mood, and functional status predicted longitudinal verbal learning performance. We hypothesized that (1) the mVLT would detect cognitive change over time, with PWH showing worse trajectories relative to controls; (2) mVLT may be more sensitive to change than a standard in-person measure of verbal learning, demonstrating incremental validity, and (3) older age would be associated with worse mVLT trajectories, particularly among PWH, consistent with accelerated/amplified cognitive aging frameworks. Within PWH, we further hypothesized that worse risk factors and comorbidities, rather than HIV-related characteristics, would predict worse mVLT trajectories.

## Materials & methods

### Participants

Participants were recruited for this study from the UC San Diego HIV Neurobehavioral Research Program (HNRP) longitudinal cohort, as well as newly recruited from the community. Follow-up mobile testing between 12 and 46 months (*M* = 26.7) post-baseline testing was conducted in a planned subset of those who completed the parent study. The follow-up data collection was supported by a one-year supplemental award to the four-year parent study, which originally collected data only at baseline. Participants were recruited into the longitudinal follow-up based on their baseline HAND and amnestic MCI status, with eligibility requiring at least one year since baseline completion, but no upper limit on the interval, which contributed to the variability in follow-up timing. Follow-up enrollment was curtailed by the 2020 COVID-19 pandemic; thus, the final analytic sample reflects participants who completed both baseline and follow-up mobile testing during the funded study window rather than attrition from the parent cohort.

The final sample with both baseline and follow-up data included 24 persons with HIV (PWH) and 13 HIV-negative control participants, ages 51–74. All participants provided written, informed consent. PWH were required to have confirmed HIV infection and to be medically stable and engaged in routine care. Exclusion criteria followed standard HNRP procedures and included neurological disorders unrelated to HIV (e.g., stroke, traumatic brain injury with >30 min loss of consciousness), severe psychiatric illness (e.g., schizophrenia), active substance dependence, or medical conditions likely to confound neurocognitive testing. HIV-negative controls met identical criteria and had confirmed seronegative status. One PWH was excluded from analyses involving standard neuropsychological outcomes due to acute intoxication on the day of their in-person study visit, supplemented by urine toxicology.

### Design and procedure

#### Neuromedical assessment

A standard HNRP neuropsychological battery [which included the Hopkins Verbal Learning Test-Revised; HVLT-R (21) using alternate forms] and neuromedical assessment were completed within six months of each corresponding mVLT burst. HIV disease characteristics (documented HIV serostatus, CD4+ T-cell count, and plasma viral load, dichotomized as detectable vs. undetectable) were verified through routine HNRP procedures.

#### Mobile verbal learning test (mVLT)

The mVLT is a brief, smartphone-delivered measure of verbal learning developed and validated by Moore et al. (17). A modified version of this test is distributed by the NeuroUX mobile cognitive assessment platform (18, 19). At each timepoint (baseline and follow-up), participants completed a 14-day remote mobile mVLT burst. Participants were paid for their participation (see (17) for details). As in Moore et al. (17), participants were provided loaner Android smartphones during their in-person study visit preloaded with the study app and received instructions and completed a training session. Next, they completed one daily mVLT session per day for 14 days, administered remotely and unsupervised at counterbalanced times across morning, afternoon, and evening. Fourteen unique 12-word lists of semantically unrelated nouns were used, matched on word frequency and constrained to comparable lexical characteristics (e.g., word length, part of speech) (17). The same fixed list order was repeated at follow-up to avoid introducing new list-learning variance.

In each daily session, participants viewed 12 words simultaneously for 30 s, after which the words disappeared, and participants provided spoken free-recall responses. Each daily session included three learning trials (Trials 1–3), with responses audio-recorded for scoring and later transcribed and scored by trained research assistants. Trials were excluded when they were considered invalid (e.g., interruptions during recall). Participants completed 14 daily sessions at each timepoint, yielding data on up to 42 learning trials per participant per burst. The mean interval between baseline and follow-up mVLT timepoints was 26.7 months (*SD* = 9.5 months).

#### mVLT outcome variables

Primary mVLT outcomes were computed separately at each timepoint (baseline and two-year follow-up) using data from the 14 daily mVLT sessions completed within each assessment burst. Our primary outcome was Aggregate Mean Total Recall (henceforth Aggregate Mean), defined as the average number of correctly recalled words per day (summed across Trials 1–3). As each daily session used a unique 12-word list, aggregate scores therefore reflect performance averaged across 14 distinct word lists within each timepoint. Aggregate Mean was pre-specified as the primary outcome because averaging performance across 14 daily administrations with distinct word lists provides a more stable and reliable estimate of verbal learning efficiency than any single-session metric, minimizing the influence of day-to-day variability and transient factors such as fatigue or distraction (13, 16). Aggregate Median Total Recall was also examined to assess robustness to non-normality; because it was highly correlated with Aggregate Mean, it was not analyzed further. Four secondary outcomes provided additional characterization of learning performance. Best Total Recall (henceforth Best Score) captured the highest total recall score observed on any single day (summed across Trials 1–3) within the 14-day burst. First-Administration reflected total recall on the first completed session (i.e., first day) at each timepoint. Variability captured the within-person standard deviation of daily total recall scores (summed across Trials 1–3) across the 14-day burst. Learning Slope was computed separately within each timepoint as the individual-level linear slope of daily total recall across study day within the 14-day burst (i.e., slope coefficient for Day), requiring 3 or greater completed days. Because the mVLT involves repeated daily administrations within each testing burst, we conducted secondary analyses modeling within-burst learning trajectories to evaluate whether group differences in aggregate performance could be attributed to differential daily practice effects.

### Standard neuropsychological assessment

The HVLT-R is a widely used, well-validated measure of verbal learning and episodic memory that served as the gold-standard comparator in the present study. Standard HVLT-R learning and recall indices have demonstrated particular sensitivity to HAND (22), with large normative datasets supporting its use across clinical and research settings (21). The HVLT-R includes six alternate forms, each containing 12 words from three semantic categories. Performance is indexed by Total Learning (sum of Trials 1–3) and Delayed Recall after a 20–25 min interval. We examined both raw scores and practice-adjusted scaled scores, which correct for expected practice effects using large HNRP longitudinal regression-based norms (23). Because participants in the HNRP cohort enter research at different stages of clinical care and may have accrued varying durations of participation in the parent study, prior HVLT-R exposure varied substantially. Before the neuropsychological assessment paired with the baseline mVLT burst, PWH had previously completed an average of 6.1 HVLT-R administrations (*SD* = 6.2; range = 0–27), whereas controls had completed an average of 1.5 administrations (*SD* = 1.6; range = 0–4), a significant difference (*p* = .01).

Visual learning and memory was assessed using the Brief Visuospatial Memory Test-Revised (BVMT-R), in which participants are asked to learn and reproduce a set of geometric designs across three learning trials, followed by a 20–25 min delayed recall. To obtain a more stable index of domain-general learning performance, we derived a Learning Composite score by averaging the T-scores from the BVMT-R Total Learning and the HVLT-R Total Learning.

Multiple clinical and behavioral assessments were administered at the time of each HNRP assessment and included the Charlson Comorbidity Index, a validated weighted summary of 19 medical conditions assessed via clinician interview, medical record review, and laboratory data (dementia item excluded) (24); Framingham Cerebrovascular Disease Risk Score (25); three domains of the Medical Outcomes Study-HIV Health Survey (self-reported Social, Cognitive, and Physical Functioning) (26); and the Beck Depression Inventory–II (BDI-II), assessing current self-reported depressive symptoms.

### Statistical analysis

All analyses were conducted in R (v4.2). Baseline group differences were evaluated using independent samples *t*-tests and *χ*² or Fisher's Exact tests, as appropriate.

#### Aim 1: longitudinal mVLT and HVLT performance

For each cognitive outcome, we fit linear mixed-effects models (LMMs; *lmer* package) with fixed effects of Group (PWH vs. Controls), Timepoint (Baseline vs. Follow-up), and their interaction. Models additionally included baseline age, years of education, sex, and inter-assessment interval (months) as covariates; continuous covariates were mean-centered. Participant-specific random intercepts were included, without random slopes due to convergence limitations. Degrees of freedom were estimated using the Satterthwaite approximation (*lmerTest* package). Significant interactions were probed using estimated marginal means (*emmeans* package). *Sensitivity Analyses:* Two complementary sensitivity analyses were conducted to evaluate the robustness of longitudinal effects, and to examine whether within-burst learning dynamics contributed to observed group differences, respectively. *(1) Time as a continuous variable:* to account for variability in the interval between baseline and follow-up, mVLT models were refit with time modeled continuously as months since baseline. *(2) Within-burst daily learning trajectories.* To directly model within-burst practice effects, daily mVLT performance was analyzed using LMMs with fixed effects of Day (centered), Group, Timepoint, and their interactions, with participant-specific random intercepts, random slope for Day, and the same covariates as in Aim 1. This tested whether group differences in aggregate performance could be attributed to differential daily learning rates within testing bursts.

Associations between mVLT Aggregate Mean change and HVLT-R change scores were examined using Pearson bivariate correlations.

#### Aim 2: Age moderation and baseline predictors of longitudinal change

*Aim 2a*: To test whether age moderated longitudinal mVLT change as a function of HIV status, we fit LMMs including Group × Timepoint × Age interactions for each mVLT outcome, adjusting for the same covariates as in Aim 1. *Aim 2b*: Within PWH, baseline clinical and health-related predictors of longitudinal mVLT change were examined using LMMs with Timepoint × Predictor interactions. To limit the number of statistical comparisons, Aim 2b analyses were restricted to the Aggregate Mean (primary outcome) and were considered exploratory.

## Results

### Sample characteristics

PWH and controls were not formally age-matched but did not significantly differ in age at baseline (PWH: *M* = 60.4; *SD* = 5.7; Controls: *M* = 61.2; *SD* = 6.2), sex distribution, racial/ethnic composition, or test-retest interval (Table 1). Controls had significantly more years of education than PWH, and PWH reported higher depressive symptoms and had higher comorbidity burden on the Charlson Comorbidity Index (*p*s < .05). Among PWH, nearly all were on ART and virally suppressed, with high current CD4 counts and long-standing HIV infection. Full descriptive statistics are presented in Table 1.

**Table 1:** Sample characteristics.

Variable	PWH (*n* = 24)	Controls (*n* = 13)	
	M(SD) or *n* (%)	M(SD) or *n* (%)	*p*
Age (years)	60.4 (5.7)	61.2 (6.2)	.685
	range: 51–73	range: 52–71	
Education (years)	13.8 (3)	15.9 (2.3)	.023*
Sex (male), *n* (%)	19 (79%)	7 (54%)	.143
Race/ethnicity, (n, %)			.584
Non-Hispanic White	16 (66.7%)	10 (76.9%)	
Hispanic/Latino(a)	5 (20.8%)	1 (7.7%)	
African American/Black	3 (12.5%)	2 (15.4%)	
Test-retest interval (months)	26.1 (10.2)	27.7 (8.4)	.611
BDI-II	10.5 (10.2)	4.4 (3.6)	.012*
Framingham CVD Risk Score	20.8 (14.1)	21.2 (17.1)	.943
Charlson Comorbidity Index	7.4 (4.8)	2.7 (2.9)	.001*
HIV characteristics			
HIV duration (years)	22.4 (7.6)		
CD4 (nadir)	135.1 (173)		
CD4 (current)	741.6 (213.4)		
On ART, *n* (%)	23 (96%)		
Undetectable viral load, *n* (%)	23 (96%)		
MOS-HIV Physical Function	70.6 (17.6)		
MOS-HIV Social Function	76.2 (29.4)		
MOS-HIV Cognitive Function	76.7 (22.0)		

* *p* < .05; BDI, beck depression inventory; ART, antiretroviral therapy; CVD, cardiovascular disease; MOS, medical outcome study-HIV health survey.

### Aim 1: group differences in longitudinal learning performance

Full model output for mVLT and HVLT outcomes is shown in Supplementary Tables S1, S2 and un-adjusted group means and standard deviations appear in Supplementary Table S3.

#### mVLT

**Figure 1:**
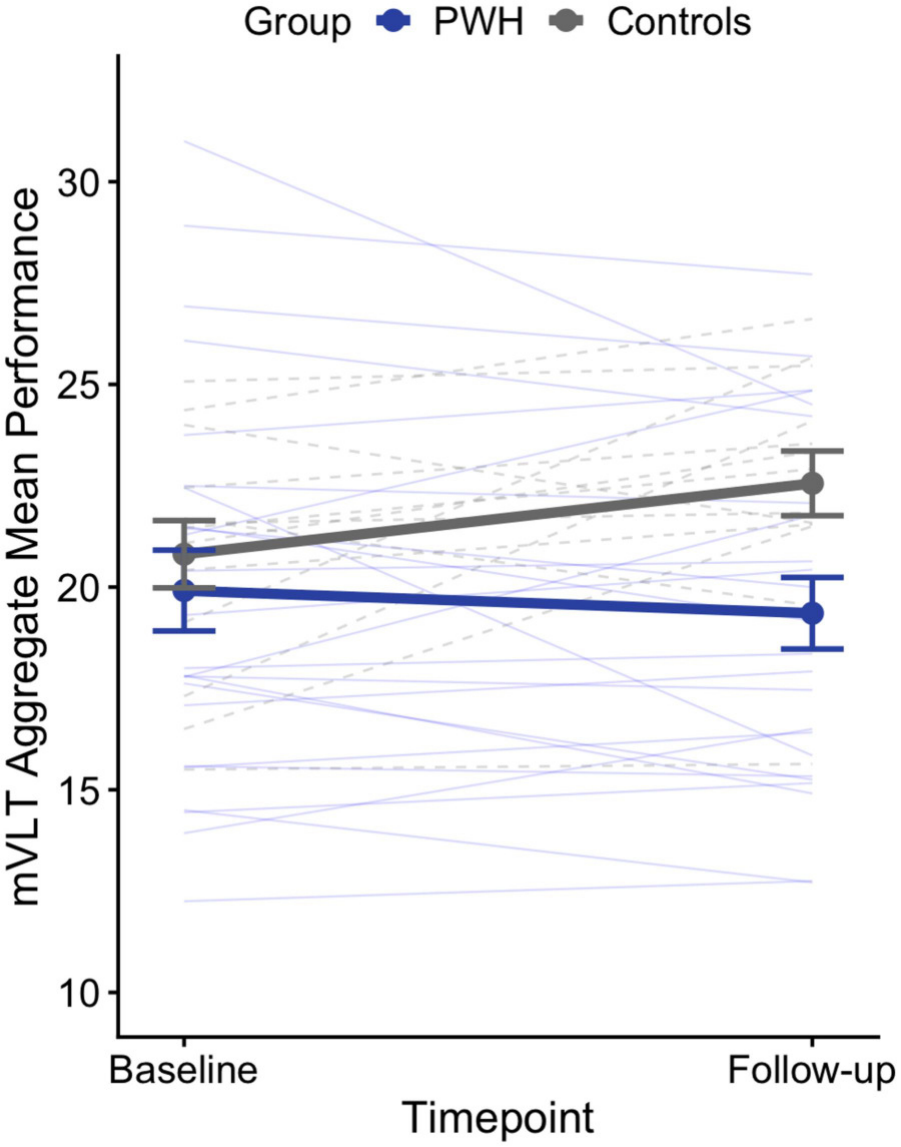
Longitudinal trajectories of mVLT Aggregate mean performance in PWH and controls. Thin lines represent individual participants with controls represented by dashed lines; bold lines and error bars represent group means ± SE.

A significant effect of education was observed for Aggregate Mean, First Administration, and Best Score (*p*s < .05), and a significant effect of baseline to follow-up assessment interval was observed for all outcomes except for Learning Slope (*p*s < .05). A main effect of Timepoint was significant for Aggregate Mean [*B* = 1.75; *SE*(*B*) = 0.72; *p* = .020] and First Administration [*B* = 4.38; *SE*(*B*) = 1.62; *p* = .010], such that there was overall improvement over time across groups. A significant main effect of Group was observed for Variability, such that PWH showed overall less variable performance than controls [*B* = −0.90; *SE*(*B*) = 0.41; *p* = .030]. A significant Group × Timepoint interaction was observed for Aggregate Mean [*B* = −2.31; *SE*(*B*) = 0.89; *p* = .014; Figure 1]. Simple slopes analyses demonstrated that controls showed significant improvement in aggregate mean performance over time (*B* = 1.75; *SE* = 0.74, *p* = .023), whereas PWH showed no significant change at the group level (*B* = −0.56; *SE* = 0.55, *p* = .311). Individual trajectories (Figure 1) indicated heterogeneity in within-person change among PWH, including decreases in aggregate performance in a subset of participants. The Group × Timepoint interaction for First-Administration trended in the same direction but did not reach significance [*B* = 3.80; *SE*(*B*) = 2.02; *p* = .067]. No significant interactions were observed for Best Score, Variability, or Learning Slope (*p*s ≥ .110).

#### mVLT (sensitivity analyses)

*(1) Time as a continuous variable.* Modeling time continuously yielded results that were largely consistent with primary findings. Notably, the Group × Timepoint interaction for mVLT Aggregate Mean remained significant [*B* = –.08; *SE*(*B*) = 0.03; *p* = .018]. *(2) Within-burst daily learning trajectories*. Day-level LMMs revealed a significant main effect of Timepoint, such that daily mVLT performance was higher at follow-up relative to baseline across groups [*B* = 1.63; *SE*(*B*) = 0.41; *p* < .001]. The Group × Timepoint interaction was significant [*B* = −2.15; *SE*(*B*) = 0.51; *p* < .001], indicating that PWH showed significantly less improvement from baseline to follow-up compared to controls when daily performance was averaged across the testing burst. There was also a significant Day × Timepoint interaction, such that daily performance did not change across days at baseline [*B* = 0.03; *SE*(*B*) = 0.05; *p* = .514] but showed a small but significant decrease across days at follow-up [*B* = −0.10; *SE*(*B*) = 0.05; *p* = .046]. Notably, the Day × Group × Timepoint interaction was not significant (*p* = .197), indicating that group differences in longitudinal change did not vary as a function of within-burst learning slopes. See Supplementary Table S4 for full model output. These findings suggest that longitudinal group differences in aggregate mVLT performance were not driven by short-term practice effects, supporting the use of burst-level summary metrics in primary analyses.

#### HVLT-R

No significant main effects of Group, Timepoint, or their interaction emerged for HVLT-R outcomes, in either raw or practice-adjusted scaled scores (all *p*s ≥ .21). When examining the Learning Composite, there was a significant main effect of Timepoint, such that there was an overall improvement in learning performance across groups from baseline to follow-up [*B* = 6.76; *SE*(*B*) = 1.50; *p* < .001]. The pattern of results remained the same when controlling for the number of previous HVLT-R administrations. Supplementary Figures S1, S2 plot individual HVLT trajectories.

#### Association between mVLT and HVLT mean change

In the full sample, changes in performances indexed by mVLT Aggregate Means were not significantly associated with changes on HVLT-R Learning [*r*(34) = −.18; *p* = .316], HVLT-R Delayed Recall [*r*(34) = .10; *p* = .560], or their practice-adjusted scaled score counterparts (*r*s = −.21 to.10; *p*s = .245–.574). The pattern of results did not change when controlling for the number of previous HVLT-R administrations (*p*s ≥ .180).

### Aim 2a: age moderation of longitudinal mVLT performance in PWH vs. controls

**Figure 2:**
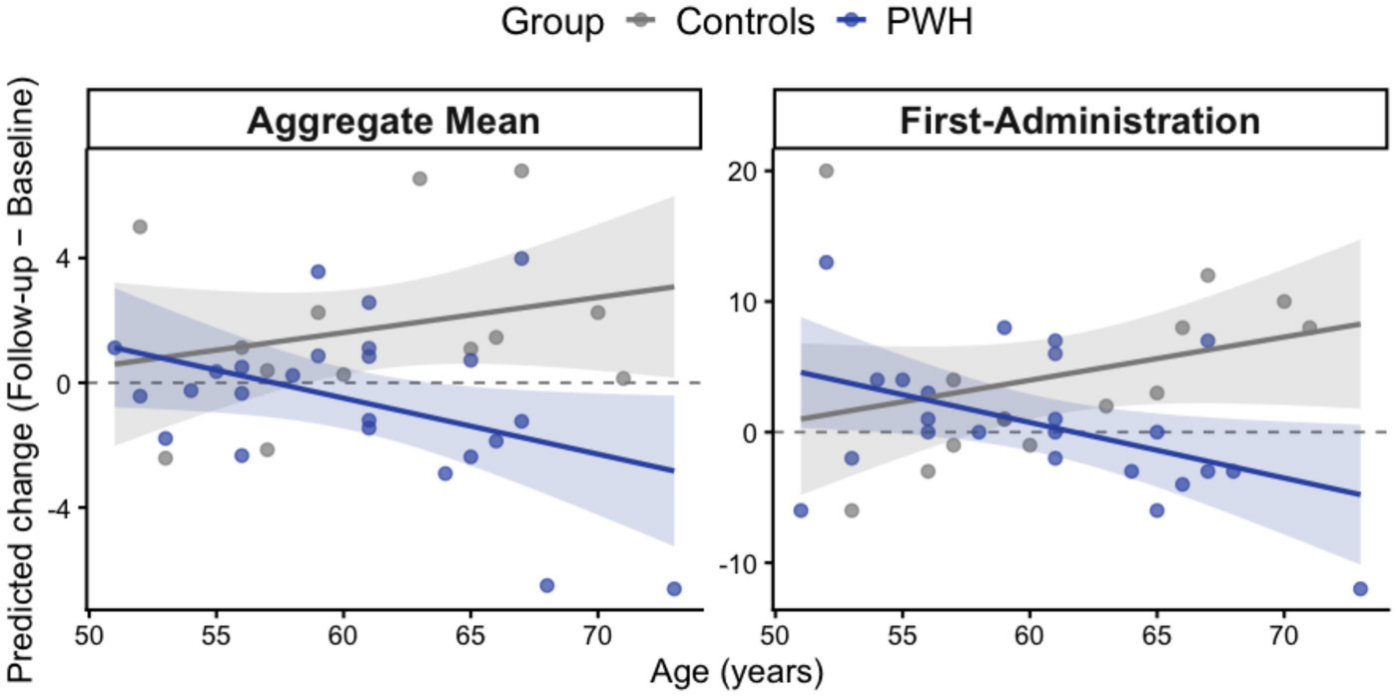
The moderating effect of age on the relationship between HIV status and longitudinal mVLT performance across several metrics. Predicted change reflects model-estimated follow-up minus baseline mVLT performance; points show observed individual change.

We next tested whether age moderated the association between HIV status and magnitude of the baseline-follow-up difference, controlling for the same covariates as in Aim 1. For the mVLT, significant Group × Timepoint × Age interactions were observed for Aggregate Mean [*B* = −0.29; *SE*(*B*) = 0.14; *p* = .049] and First Administration [*B* = −0.76; *SE*(*B*) = 0.32; *p* = .023], with a similar marginally significant trend observed for Best Score [*B* = −0.40; *SE*(*B*) = 0.20; *p* = .052]. Follow-up simple effects analyses indicated that within PWH, increasing age showed a significant or a marginal association with worse mVLT trajectories [First Administration: *B* = −0.43; *SE*(*B*) = 0.21; *p* = .049; Aggregate Mean: *B* = −0.18; *SE*(*B*) = 0.09; *p* = .065] whereas age was not significantly associated with longitudinal change in controls (*p*s ≥ .219), and trended in the opposite direction (Figure 2**)**. No significant age moderation effects were observed for Variability or Learning Slope (*p*s ≥ .526). Parallel models examining HVLT-R and Learning Composite outcomes did not reveal significant Group × Timepoint × Age interactions (*p*s ≥ .306).

### Aim 2b: predictors of longitudinal mVLT performance among PWH

**Figure 3:**
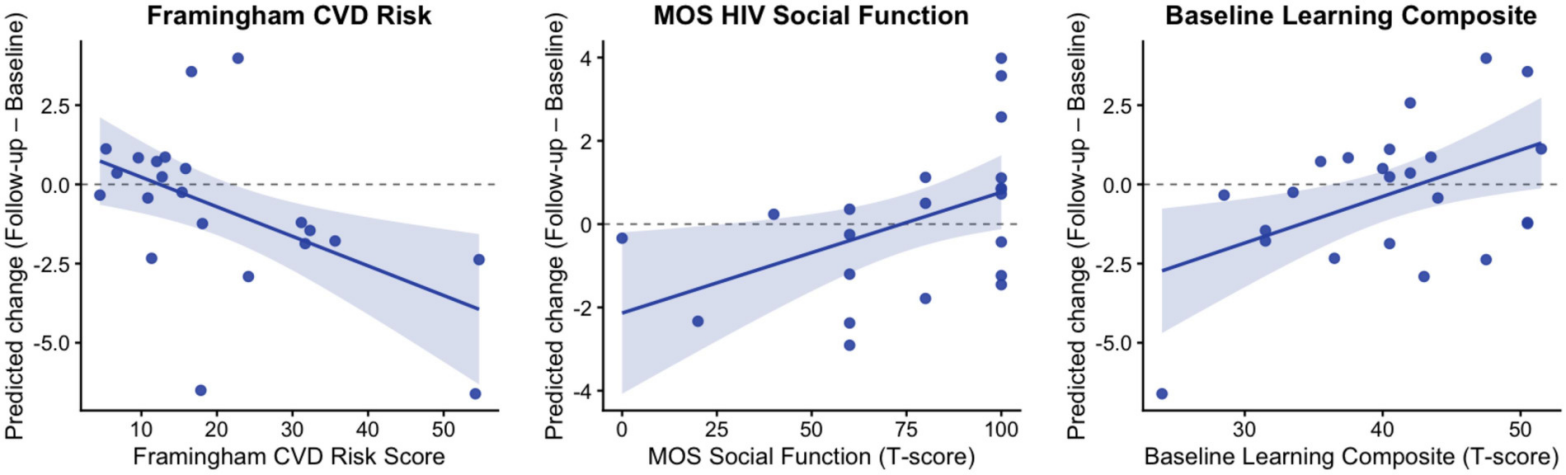
Predicted change in mVLT Aggregate mean performance (follow-up minus baseline) as a function of baseline risk factors among PWH. Solid lines depict model-predicted change from linear-mixed effects models adjusted for age, education, sex, and inter-assessment interval; shaded bands indicate 95% confidence intervals. Points represent individual observed change scores.

To identify baseline factors associated with mVLT trajectories among PWH, we fit separate covariate-adjusted LMMs examining Timepoint × Predictor interactions for mVLT Aggregate Mean, with the same covariates as in Aim 1. Interaction coefficients and model statistics for all tested predictors are presented in Table 2 and significant interactions are plotted in Figure 3. Higher baseline Framingham CVD Risk Score, lower baseline MOS-HIV Social Function, and lower baseline global learning performance (Learning Composite) were associated with worse mVLT trajectories (*p*s < .05). No significant interactions were observed for Charlson Comorbidity Index, depressive symptoms (BDI-II), estimated duration of HIV infection, current or nadir CD4, or other MOS subscales. Parallel models in controls did not reveal significant Timepoint × Predictor associations for the Charlson Comorbidity Index, Framingham CVD Risk Score, global learning performance, or depressive symptoms (*p*s ≥ .110).

**Table 2:** Timepoint × baseline predictor interactions from linear mixed effects models predicting mVLT aggregate mean change among PWH.

Timepoint × baseline predictor	B	SE(B)	df	t	*p*
Framingham CVD Risk Score	−0.09	0.03	22	−2.82	.010*
Charlson Comorbidity Index	−0.03	0.11	24	−0.28	.779
BDI-II Depressive Symptoms	−0.01	0.05	24	−0.23	.818
Learning Composite	0.15	0.06	23	2.65	.014*
MOS-HIV Cognitive Function	0.02	0.02	21	0.85	.407
MOS-HIV Physical Function	0.02	0.02	21	0.80	.430
MOS-HIV Social Function	0.03	0.01	21	2.39	.026*
CD4 nadir	0.002	0.003	24	0.49	.627
CD4 absolute	−0.002	0.002	24	−1.01	.322
Estimated duration of infection	0.10	0.07	24	1.58	.128

Each row represents the Timepoint × Predictor interaction term from a separate covariate-adjusted linear mixed-effects model. Models included fixed effects of Timepoint, the baseline predictor, and their interaction, with age, education, sex, and inter-assessment interval as covariates. Coefficients reflect the degree to which each baseline predictor moderated the magnitude of baseline-to-follow-up change in mVLT Aggregate Mean.

* *p* < .05.

## Discussion

In this prospective longitudinal study, we found that a mobile verbal learning test (mVLT) differentiated verbal learning trajectories in middle-aged to older adults living with and without HIV. When the same word lists were presented 12–46 months following baseline testing, controls exhibited the expected improvement over time (practice effects), whereas persons with HIV (PWH) did not. Notably, group differences were evident in longitudinal trajectories (i.e., practice-related gains) rather than performance level at a single timepoint, suggesting that the mVLT may be particularly sensitive to HIV-related differences in learning dynamics rather than cross-sectional impairment. This pattern was most evident when learning was summarized across repeated administrations using aggregate mean scores, which emerged as the most sensitive indicator of group differences in longitudinal trajectories. Notably, substantial inter-individual variability was present in PWH, such that despite the absence of group-level change and the absence of normative improvement, a subset of PWH showed within-person decreases in mVLT performance over time. Moderation analyses indicated that among PWH, older age, higher cerebrovascular risk, poorer social functioning, and lower baseline learning performance predicted worse mVLT trajectories. With future replication in larger samples, these preliminary findings suggest that repeated mobile cognitive assessment may be sensitive to subtle learning inefficiencies or cognitive vulnerability in PWH that could be missed by standard testing.

Consistent with our hypotheses, we found that age moderated mVLT trajectories, such that older PWH showed worse longitudinal trajectories across several metrics, whereas this was not observed in controls. These findings are consistent with accelerated or accentuated aging-related cognitive vulnerability in HIV (3, 4). Notably, other longitudinal cohort work has similarly documented greater-than-expected age effects in PWH, particularly for episodic memory (10, 27), supporting the idea that aging may amplify susceptibility in memory-related systems.

In addition, longitudinal mobile verbal learning trajectories were more strongly associated with comorbid risk factors than with traditional HIV disease markers (e.g., CD4 count, duration of infection) (9, 11, 28–30). Cerebrovascular disease risk, in particular, emerged as a significant predictor of decreased performance over time. This is consistent with mounting evidence that vascular risk is over-represented in PWH and plays a central role in shaping cognitive impairment and decline in the modern treatment era (31–33). In contrast, global medical comorbidity burden was not a significant predictor of longitudinal change. This differs from prior work that found that greater medical comorbidity burden was associated with worse neurocognitive trajectories in HIV (11). In addition to that study having a larger sample size and a longer follow-up period, another explanation is that the Charlson index represents a broad composite measure that aggregates heterogeneous conditions with potentially distinct cognitive effects. It is plausible that specific comorbidities, such as cerebrovascular disease, exert a more direct influence on verbal learning outcomes than a global summary score. The sample size limited our ability to examine individual comorbidity profiles or interactions, which may obscure effects captured in larger cohorts.

Beyond vascular comorbidities, poorer social functioning and lower baseline global learning performance were associated with worse mobile verbal learning trajectories. These findings are consistent with models of cognitive reserve (34), in which individuals with lower baseline cognitive capacity or reduced social engagement may be less able to compensate for age- or disease-related neural vulnerability. In the context of HIV, social functioning is increasingly recognized as a meaningful contributor to cognitive aging, reflecting cumulative effects of educational quality, socioeconomic resources, chronic stress, and access to cognitively enriching environments (29).

The divergence in practice-related gains has potentially important implications. Practice effects, or improved cognitive performance following repeated exposure to test materials, are increasingly recognized as a meaningful cognitive signal rather than a methodological nuisance (35, 36). Attenuated or absent practice effects have been associated with mild cognitive impairment and Alzheimer's disease, predict future cognitive decline, and relate to biomarkers of neurodegeneration (36–38). Within this framework, reduced learning gains in PWH could reflect early cognitive vulnerability that precedes overt decline, potentially related to impaired encoding or consolidation of prior experience. However, attenuated practice effects may also reflect broader reductions in learning efficiency, including diminished strategy formation, attentional consistency, or executive control processes that support learning across repeated encounters. Fronto-striatal dysfunction, a core feature of HAND (7), may limit the ability to capitalize on repeated exposure even when basic memory storage and hippocampal function is relatively preserved. Disentangling these possibilities will require integration of longitudinal cognitive trajectories with neuroimaging, biomarker data, and deep phenotyping of learning and memory profiles, particularly across longer follow-up periods that can distinguish stable differences in learning efficiency from emerging cognitive decline, given overlap between HAND and early neurodegenerative presentations (39).

Notably, sensitivity to longitudinal change in the present study was greatest when performance was aggregated across repeated administrations. That is, Aggregate Mean scores were more sensitive to group differences over time than single-session, learning slope, or best performance metrics. This supports the rationale for burst-based designs, in which repeated low-burden sampling can provide a more stable estimate of learning efficiency by reducing day-to-day variability and measurement noise. Although repeated exposure to new, semantically unrelated word lists across 14 consecutive days could introduce proactive interference or cumulative cognitive load, our day-level models indicated that between-group differences in longitudinal change were not driven by differences in short-term (within-burst) learning trajectories. This approach may be particularly well suited for detecting subtle cognitive vulnerability in PWH, where early change may be expressed as diminished learning efficiency rather than large decrements in absolute performance. For example, a large longitudinal study of virally suppressed PWH showed low rates of clinically meaningful decline but a substantial prevalence of subtle decline and sustained impairment, particularly with aging (30).

In contrast, the in-person gold-standard measure of episodic learning and memory (HVLT-R) did not reveal group differences in longitudinal trajectories. Although not statistically robust, both groups showed a numerical improvement from baseline to follow-up, and a significant improvement on the Learning Composite. Though contrary to our initial hypothesis of a strong positive correlation between change on both measures, this pattern is consistent with expected practice effects on a semantically structured list-learning task, particularly in the context of substantial prior exposure in PWH, who completed the HVLT multiple times on average before study entry. In contrast, the mVLT involved extensive repeated testing but used semantically *unrelated* words that limit the benefit of organizational strategies and place greater demands on episodic encoding. It is possible that relatively preserved improvement on the HVLT may reflect strategy-based learning and familiarity with task structure, whereas the absence of practice effects on the mVLT could reflect reduced learning efficiency when strategic support is minimized. Traditional neuropsychological tests emphasize peak performance under structured conditions, whereas mobile testing captures learning efficiency across repeated, low-burden exposures. Thus, repeated mobile testing paradigms may be particularly sensitive to subtle inefficiencies in learning. Together, these findings suggest that mobile and in-person assessments index partially distinct aspects of learning, with mobile testing preferentially capturing dynamic learning efficiency rather than static performance level.

Several limitations should be acknowledged. First, the sample size was modest, in part due to early termination of data collection related to the COVID-19 pandemic. Thus, effect-size estimates should be interpreted as preliminary and in need of replication. In addition, a lower sample size limited statistical power to examine subgroup effects (e.g., stratification by cognitive diagnosis) or to test more complex multivariate models of predictors of change. Second, the two-wave design and short follow-up period limited our ability to determine whether attenuated practice effects on the mVLT reflect stable differences in learning efficiency or progressive cognitive change. In addition, the wide range of follow-up interval (12–46 months) reflects the absence of an upper time limit for re-enrollment during the supplemental funding period. Though this heterogeneity introduces variability in the expected magnitude of practice effects and limits direct comparability across participants, we addressed this analytically by including inter-assessment interval as a continuous covariate in all models, and by conducting a sensitivity analysis modeling time continuously, which yielded consistent results. Third, direct comparison between the mVLT and HVLT-R was limited by substantial group differences in prior HVLT-R exposure before study entry, particularly among PWH, which likely reduced the interpretability of HVLT-R practice-related change over the study interval despite the use of alternate forms and practice-adjusted scaled scores. In addition, this may have contributed to the unexpected null (or even negative trending) correlations between the HVLT-R and mVLT. Fourth, although education was included as a covariate in all models, controls had significantly higher education than PWH, and statistical adjustment may not fully capture the broader construct of cognitive reserve. Unmeasured factors such as socioeconomic status and quality of education could contribute to observed group differences and represent an important direction for future work. Although repeated exposure was a strength of the design, future work could examine alternative list structures, such as repeated presentation of the same list to model learning curves (40), or adaptive testing paradigms. An additional future direction is to identify the minimum number of days of mobile testing required to create a stable estimate of aggregate performance.

Despite these limitations, with future replication the present findings highlight the promise of mobile, burst-based cognitive testing for detecting subtle cognitive vulnerability in PWH that may not be evident on standard in-person measures. By emphasizing learning performance across repeated, low-burden assessments rather than a snapshot performance in a single session, mobile testing may be particularly well suited for identifying subtle learning inefficiencies in aging clinical populations. Though currently primarily a research tool, mobile cognitive assessments are increasingly being adapted for clinical use. In clinical practice, such tools could serve multiple roles: periodic between-visit monitoring to flag early cognitive change, screening to triage patients for comprehensive neuropsychological evaluation, and complementing in-person assessment by capturing cognitive performance in daily life, which may differ meaningfully from performance under structured lab conditions. Attenuated practice effects or observed declines could prompt timely referral, and integration with electronic health records could support longitudinal cognitive surveillance. Realizing this potential will require establishing reliable change benchmarks, sensitivity and specificity data, and frameworks for integrating mobile assessments into clinical workflows. This model is already being implemented in large-scale initiatives such as ADNI-4, which incorporates remote speech-based cognitive screening as part of a longitudinal multi-tier assessment protocol (41). Future studies integrating mobile cognitive trajectories with neuroimaging, blood-based biomarkers, and digital health measures will be important for clarifying underlying mechanisms. Such approaches could determine whether individuals who show attenuated practice effects or within-person declines have profiles consistent with fronto-striatal or hippocampal dysfunction, accelerated aging, or early neurodegeneration, thereby supporting more precise risk stratification and intervention targeting.

## Data Availability

The raw data supporting the conclusions of this article will be made available by the authors, without undue reservation.
