# Supplementary material for: Mobile cognitive testing captures divergent longitudinal trajectories of verbal learning in adults with and without HIV
Source: Front Digit Health. 2026 Jun 16;8:1792496. doi: 10.3389/fdgth.2026.1792496 (PMC13314624; doi:10.3389/fdgth.2026.1792496)
Supplement: Supplementary file 1 [file Datasheet1.docx]

**Supplemental Online Material**

**Table S1.** LMM model output for mVLT outcomes (*n*=23 PWH and 13 controls)

| *mVLT outcome* | *B* | *SE(B)* | *df* | *t* | *p* |
| --- | --- | --- | --- | --- | --- |
| **Aggregate Mean** |  |  |  |  |  |
| Group (PWH) | 0.56 | 1.38 | 46 | 0.41 | .687 |
| Timepoint (follow-up) | 1.75 | 0.72 | 37 | 2.43 | .020* |
| Age | 0.09 | 0.1 | 37 | 0.92 | .364 |
| Education | 0.49 | 0.21 | 37 | 2.29 | .028* |
| Sex (female) | 0.56 | 1.33 | 37 | 0.42 | .674 |
| Interval | 0.13 | 0.06 | 37 | 2.23 | .032* |
| Group × Timepoint | -2.31 | 0.89 | 37 | -2.58 | .014* |
| **Best Score** |  |  |  |  |  |
| Group (PWH) | -0.44 | 1.52 | 51 | -0.29 | .773 |
| Timepoint (follow-up) | 1.23 | 1.00 | 37 | 1.23 | .225 |
| Age | 0.05 | 0.11 | 37 | 0.49 | .629 |
| Education | 0.52 | 0.23 | 37 | 2.27 | .029* |
| Sex (female) | -0.03 | 1.41 | 37 | -0.02 | .982 |
| Interval | 0.16 | 0.06 | 37 | 2.63 | .012* |
| Group × Timepoint | -1.02 | 1.24 | 37 | -0.83 | .414 |
| **First Administration** |  |  |  |  |  |
| Group (PWH) | 1.55 | 1.97 | 60 | 0.78 | .436 |
| Timepoint (follow-up) | 4.38 | 1.62 | 37 | 2.70 | .010* |
| Age | 0.05 | 0.13 | 37 | 0.37 | .717 |
| Education | 0.63 | 0.28 | 37 | 2.27 | .029* |
| Sex (female) | 2.1 | 1.72 | 37 | 1.22 | .232 |
| Interval | 0.16 | 0.08 | 37 | 2.05 | .047* |
| Group × Timepoint | -3.80 | 2.02 | 37 | -1.88 | .067^ |
| **Variability** |  |  |  |  |  |
| Group (PWH) | -0.90 | 0.41 | 69 | -2.22 | .030* |
| Timepoint (follow-up) | -0.65 | 0.39 | 37 | -1.64 | .109 |
| Age | -0.01 | 0.02 | 37 | -0.39 | .697 |
| Education | -0.02 | 0.05 | 37 | -0.32 | .748 |
| Sex (female) | -0.52 | 0.33 | 37 | -1.58 | .123 |
| Interval | 0.04 | 0.01 | 37 | 2.43 | .020* |
| Group × Timepoint | 0.8 | 0.49 | 37 | 1.64 | .109 |
| **Learning Slope** |  |  |  |  |  |
| Group (PWH) | -0.04 | 0.13 | 74 | -0.32 | .747 |
| Timepoint (follow-up) | -0.22 | 0.14 | 74 | -1.60 | .114 |
| Age | -0.003 | 0.01 | 74 | -0.44 | .658 |
| Education | 0.001 | 0.02 | 74 | 0.03 | .973 |
| Sex (female) | -0.05 | 0.10 | 74 | -0.50 | .622 |
| Interval | -0.01 | 0.004 | 74 | -1.48 | .143 |
| Group × Timepoint | 0.12 | 0.17 | 74 | 0.71 | .480 |

*Note:* Reference categories are Controls (Group), Baseline (Timepoint), and Male (Sex). Continuous predictors are mean-centered. * *p* < .05; ^ *p* <.10

**Table S2.** LMM model output for HVLT-R outcomes (*n*=22 PWH and 12 controls)

| *HVLT-R outcome* | *B* | *SE(B)* | *df* | *t* | *p* |
| --- | --- | --- | --- | --- | --- |
| **Learning (raw)** |  |  |  |  |  |
| Group (PWH) | 0.01 | 1.97 | 49 | 0.01 | .996 |
| Timepoint (follow-up) | 1.27 | 1.00 | 34 | 1.27 | .212 |
| Age | -0.02 | 0.13 | 34 | -0.13 | .901 |
| Education | 0.66 | 0.31 | 34 | 2.17 | .037* |
| Sex (female) | -0.29 | 1.73 | 34 | -0.16 | .870 |
| Interval | -0.06 | 0.08 | 34 | -0.71 | .485 |
| Group × Timepoint | 0.73 | 1.68 | 34 | 0.43 | .668 |
| **Delayed Recall (raw)** |  |  |  |  |  |
| Group (PWH) | 0.43 | 0.87 | 52 | 0.49 | .625 |
| Timepoint (follow-up) | 0.05 | 0.48 | 34 | 0.09 | .926 |
| Age | -0.03 | 0.06 | 34 | -0.55 | .589 |
| Education | 0.32 | 0.13 | 34 | 2.45 | .020* |
| Sex (female) | 0.02 | 0.74 | 34 | 0.03 | .978 |
| Interval | -0.01 | 0.04 | 34 | -0.15 | .882 |
| Group × Timepoint | 0.54 | 0.82 | 34 | 0.66 | .514 |
| **Learning (ss)** |  |  |  |  |  |
| Group (PWH) | 0.14 | 1.12 | 51 | 0.13 | .899 |
| Timepoint (follow-up) | 0.75 | 0.61 | 34 | 1.23 | .227 |
| Age | 0.002 | 0.07 | 34 | 0.02 | .981 |
| Education | 0.40 | 0.17 | 34 | 2.34 | .025* |
| Sex (female) | 0.02 | 0.97 | 34 | 0.02 | .983 |
| Interval | -0.04 | 0.05 | 34 | -0.91 | .369 |
| Group × Timepoint | 0.13 | 1.03 | 34 | 0.12 | .904 |
| **Delayed recall (ss)** |  |  |  |  |  |
| Group (PWH) | 0.73 | 1.01 | 54 | 0.72 | .478 |
| Timepoint (follow-up) | 0.27 | 0.60 | 34 | 0.46 | .651 |
| Age | -0.05 | 0.07 | 34 | -0.82 | .420 |
| Education | 0.37 | 0.15 | 34 | 2.43 | .021* |
| Sex (female) | -0.10 | 0.86 | 34 | -0.11 | .909 |
| Interval | -0.02 | 0.04 | 34 | -0.4 | .693 |
| Group × Timepoint | 0.44 | 1.01 | 34 | 0.43 | .668 |
| **Learning Composite (T)** |  |  |  |  |  |
| Group (PWH) | 1.82 | 2.96 | 50 | 0.61 | .542 |
| Timepoint (follow-up) | 6.76 | 1.50 | 35 | 4.51 | <.001* |
| Age | 0.03 | 0.20 | 35 | 0.16 | .870 |
| Education | -0.37 | 0.46 | 35 | -0.81 | .426 |
| Sex (female) | -5.43 | 2.62 | 35 | -2.07 | .046* |
| Interval | -0.01 | 0.13 | 35 | -0.09 | .927 |
| Group × Timepoint | -0.84 | 2.56 | 35 | -0.33 | .744 |

*Note:* Reference categories are Controls (Group), Baseline (Timepoint), and Male (Sex). Continuous predictors are mean-centered. * *p* < .05.

**Table S3.** Un-adjusted baseline and follow-up learning performance for PWH and controls

|  | PWH (*n*=24) | | | | | | Controls (*n*=13) | | | | | |
| --- | --- | --- | --- | --- | --- | --- | --- | --- | --- | --- | --- | --- |
|  | Baseline | | Follow-up | | ∆ | | Baseline | | Follow-up | | ∆ | |
|  | *M* | *SD* | *M* | *SD* | *M* | *SD* | *M* | *SD* | *M* | *SD* | *M* | *SD* |
| **mVLT** |  |  |  |  |  |  |  |  |  |  |  |  |
| Aggregate Mean | 19.9 | 4.9 | 19.4 | 4.3 | -0.6 | 2.6 | 20.8 | 3.0 | 22.6 | 2.9 | 1.7 | 2.9 |
| First Administration | 18.8 | 5.9 | 19.4 | 6.5 | 0.6 | 5.4 | 19.4 | 6.3 | 23.8 | 3.4 | 4.4 | 7.1 |
| Best Score | 24.3 | 5.1 | 24.5 | 5.3 | 0.2 | 3.4 | 26.2 | 3.3 | 27.4 | 3.0 | 1.2 | 4.2 |
| Variability | 3.0 | 0.9 | 3.1 | 1.0 | 0.2 | 1.1 | 3.8 | 2.1 | 3.1 | 0.8 | -0.6 | 1.9 |
| Learning Slope | 0.0 | 0.3 | -0.1 | 0.4 | -0.1 | 0.4 | 0.1 | 0.5 | -0.2 | 0.4 | -0.2 | 0.7 |
| **HVLT** |  |  |  |  |  |  |  |  |  |  |  |  |
| Total Learning (raw) | 24.8 | 5.9 | 26.1 | 5.7 | 1.3 | 5.5 | 26.1 | 3.6 | 28.1 | 4.0 | 2.0 | 3.3 |
| Total Learning (ss) | 8.5 | 3.3 | 9.3 | 3.4 | 0.8 | 3.2 | 9.5 | 1.9 | 10.4 | 2.6 | 0.9 | 2.4 |
| Delayed Recall (raw) | 8.5 | 2.4 | 8.5 | 2.8 | 0 | 2.5 | 9.6 | 1.7 | 10.2 | 1.8 | 0.6 | 2.0 |
| Delayed Recall (ss) | 8.4 | 2.5 | 8.7 | 3.3 | 0.3 | 3.0 | 9.8 | 2.1 | 10.5 | 2.4 | 0.7 | 2.6 |

*Note:* Values are unadjusted descriptive statistics. Inference is based on covariate-adjusted linear mixed-effects model. ss = practice-adjusted scaled score; Change is defined as follow-up minus baseline score. HVLT analyses include 22 PWH and 12 controls.

**Table S4.** LMM model output of daily-level mVLT performance across study days by group and timepoint

| *Model term* | *B* | *SE(B)* | *df* | *t* | *p* |
| --- | --- | --- | --- | --- | --- |
| Day | 0.04 | 0.08 | 120 | 0.52 | .607 |
| Group | 0.56 | 1.32 | 40 | 0.42 | .674 |
| Timepoint | 1.63 | 0.41 | 785 | 3.94 | <.001* |
| Age | 0.12 | 0.1 | 37 | 1.23 | .227 |
| Education | 0.48 | 0.21 | 38 | 2.3 | .027* |
| Sex | 0.81 | 1.3 | 37 | 0.63 | .536 |
| Interval | 0.15 | 0.06 | 37 | 2.56 | .015* |
| Day × Group | -0.02 | 0.1 | 119 | -0.17 | .868 |
| Day × Timepoint | -0.22 | 0.1 | 798 | -2.07 | .039* |
| Group × Timepoint | -2.15 | 0.51 | 788 | -4.19 | .001* |
| Day × Group × Timepoint | 0.17 | 0.13 | 796 | 1.29 | .197 |

*Note:* Reference categories are Controls (Group), Baseline (Timepoint), and Male (Sex). Continuous predictors are mean-centered. * *p* < .05.

**Figure S1.** Longitudinal trajectories of HVLT-R Learning (raw) performance in PWH and controls


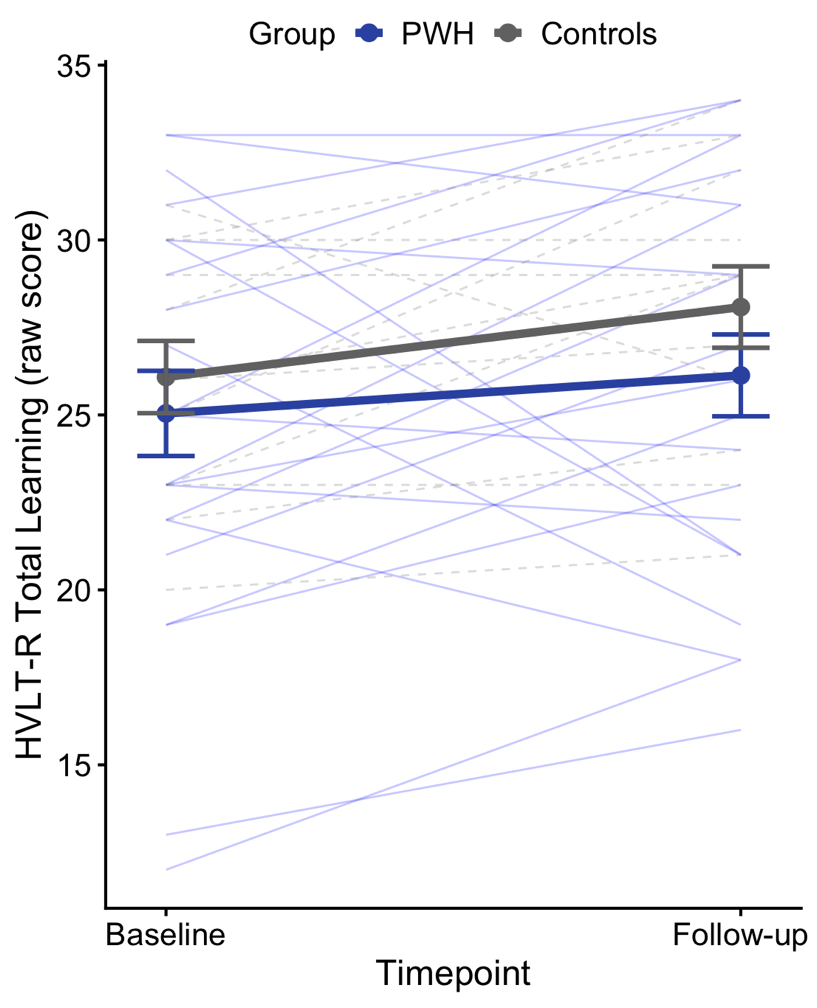


*Note:* Thin lines represent individual participants with controls represented by dashed lines; bold lines and error bars represent group means ± SE.

**Figure S2.** Longitudinal trajectories of HVLT-R Delayed Recall (raw) performance in PWH and controls

**
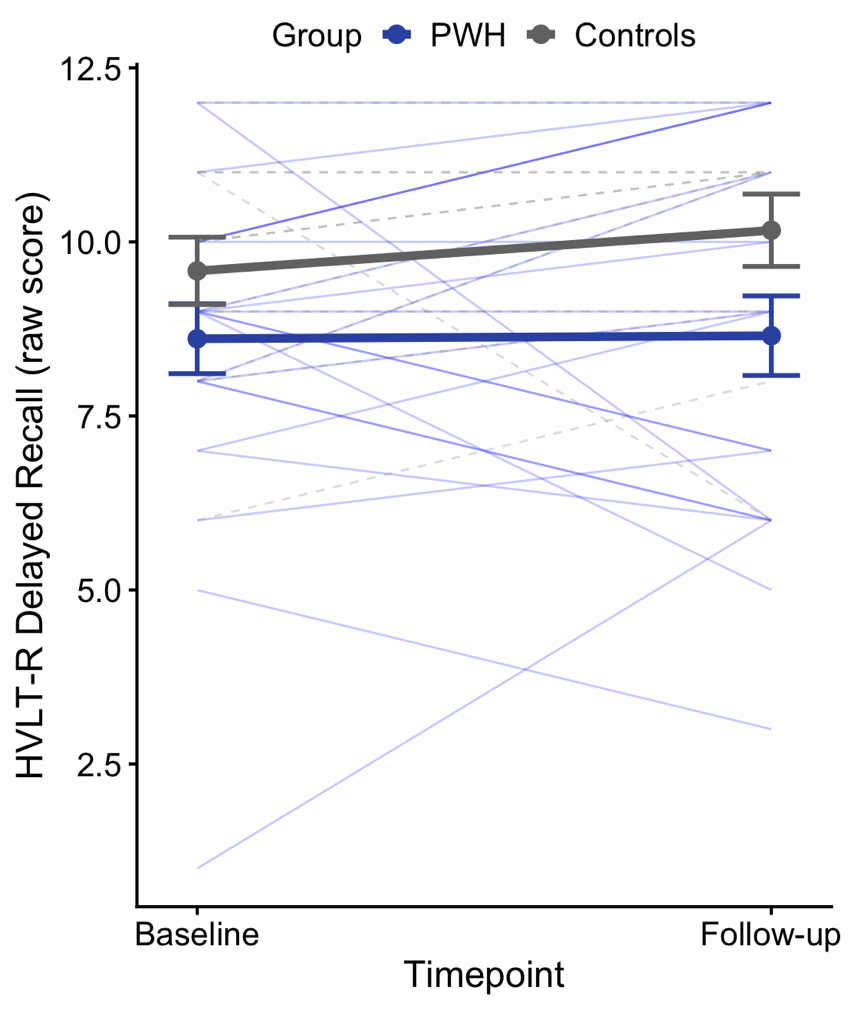
**

*Note:* Thin lines represent individual participants with controls represented by dashed lines; bold lines and error bars represent group means ± SE.
